# The Poor Man's Home.—XXIV

**Published:** 1898-02-05

**Authors:** 


### Modern Sociology.

### THE POOR MAN'S HOME.—XXIV.

Model Lodging-houses (*continued*).

When the Glasgow Improvement Trust first began their operations, they found that many of the houses they were about to destroy were occupied not only by families, but by lodgers. Lodgers were found in 14 per cent. of the one-room houses, in 23 per cent. of the two-room, and in 32 per cent. of the three-room dwellings in the city. It was manifestly both cruel and foolish to turn these people out on the streets without making any provision for their housing. If that were done, the only result would be to send them to overcrowd the lodging-houses which were suffered to exist, and to make these as bad as any that were destroyed. As the terms of the Trust permitted the trustees to provide accommodation for those whom they dispossessed, they set about building not only houses for the families whom they turned out, but improved houses also for the lodgers. The first corporation model lodging-house was completed on February 6th, 1871, and the experiment proved so successful that it was proceeded with, and there are now six lodging-houses for men and one for women.

The experiment has been a success from every point of view. It has given the homeless and more or less vagrant population better accommodation than they could ever command before; it has shown the way to philanthropists who were desirous of helping the poorest of the poor; and it has proved that even these of the poorest class can get decent, healthy, and fairly comfortable housing at a rate which they can easily pay, and yet give a good return on the money invested in these lodgings. From the first these lodging-houses have been a financial success.

Their chief superiority over the old lodging-houses lies in the fact that each lodger has complete privacy. The arrangement of the beds is such as to give this and yet economise space. Two beds are placed one over the other, somewhat after the fashion of bunks on board ship, save that the occupant of the lower bunk enters his cubicle from one side, and a partition, rising from the front of his bed at the top, forms the back of the upper berth, which is entered from a cubicle on the other side. A wire netting stretched over the top of the cubicles prevents any communication between the occupants; and the thefts of clothing, &c., which were so common in the old lodging-houses, are practically impossible in these. While, as we have shown, space is economised, there is an ample supply of air, the allowance being 400 cubic feet for each occupant. Besides the bedrooms, divided into rows of cubicles in the manner we have described, there are reading-rooms, dining-rooms, baths, and kitchens where the lodgers can cook their own food.

The charge for a night's lodging in any of these houses is 3d. to 3½d. for women, and 3½d. to 4½d. for men. The extra penny entitles the occupant to an extra sheet. At this moderate rate of payment the model lodging-houses return more than 5¼ per cent. on the original cost. Apart from financial considerations, the result has been equally gratifying. Infectious disease, when it has appeared, has been stamped out with a promptness impossible under the old system. During a recent epidemic of small-pox the municipality granted a week's free lodging to anyone who would be vaccinated. This precautionary measure was laughed at by some London papers, as savouring of "grandmotherly" care; but the will and the power to take such measures must tend largely to prevent the spread of infectious disease. These lodging-houses have also lessened the number of persons lodging in private families, which, in the small homes of the poor, is always objectionable. Moreover, the keepers of private lodging-houses, finding that they had to compete with the superior accommodation of the corporation, have been compelled to raise their standard of comfort. It is probable, however, that they get only those whose habits are such as to make them resent the very mild demands in the way of conduct which are insisted on in the municipal houses.

The corporation has, however, one competitor in the work who is worthy of description. Mr. Robert Burns came to Glasgow as manager of the municipal lodging-houses. In that post he was very successful, and his experience in them led him to think that it was possible yet further to increase the comfort of the accommodation, and yet earn a substantial profit. This he has succeeded in doing, and the seven lodging-houses he owns are among the most interesting things in Glasgow. Instead of going to the expense of building houses, he has been fortunate in getting disused warehouses in some of the busiest parts of the city, and has reconstructed them to suit his purpose. The prices are the same as in the city lodging-houses; but experience has shown where improvements could be wrought. The cubicles are arranged on the same principle as we have described. On each storey there is a lavatory, with hot and cold water, a bath, and a water-closet intended for night use only. The closets for day use are on the ground floor, where are also to be found a sitting and eating room, a hall, where religious meetings are held on Sundays, and free entertainments on occasional week-nights, lavatories, and foot-baths. These foot-baths are very popular. They are arranged round one side of the lavatory, and men who have been on tramp for a whole day are often glad to soak their feet in warm water, even though they do not desire a complete bath, which, however, if they want it, can be had free of charge. In the sitting-rooms the men can take their meals, read, and smoke. Newspapers are lying about, and games of various kinds are provided free. In the sitting-rooms lockers are provided, where each lodger can keep his belongings. A deposit of 6d. is required for the key of this locker. In each house there is a store, where provisions can be bought in smaller quantities than the shops will retail them at, but which are sold at a very cheap rate. In the kitchen the men can cook their own meals. A large range shaped like a horse-shoe runs round the kitchen. In some of Mr. Burns's homes the range is large enough to permit of 400 persons cooking their meals at the same time. Cooking utensils and plates are provided free of charge, and are washed by attendants kept in the place for that purpose. Wash-basins are at hand, so that any inmate who is sufficiently fastidious to desire to wash his hands before eating has every facility for doing so, and cleanliness is encouraged in every way.

